# Numerical analysis of the MASnI_3_/CZT(Se_1−*x*_S_*x*_) interface to boost the performance *via* band offset engineering

**DOI:** 10.1039/d5ra02248g

**Published:** 2025-05-20

**Authors:** Rasmiah S. Almufarij, Muazma Jamil, M. Yasir Ali, M. D. Alshahrani, Salhah Hamed Alrefaee, Mohamed Abdelsabour Fahmy, Islam Ragab, A. R. Abd-Elwahed, Adnan Ali, Arslan Ashfaq

**Affiliations:** a Department of Chemistry, College of Science, Princess Nourah bint Abdulrahman University P. O. Box 84428 Riyadh 11671 Saudi Arabia; b Department of Physics, Government College University Faisalabad 38000 Pakistan adnnan_1982@yahoo.com; c Department of Physics, College of Science, University of Bisha P. O. Box 551 Bisha 61922 Saudi Arabia; d Department of Chemistry, College of Science, Taibah University Yanbu-30799 Madinah Saudi Arabia; e Adham University College, Umm Al-Qura University Adham 28653 Makkah Saudi Arabia; f Faculty of Computers and Informatics, Suez Canal University New Campus 41522 Ismailia Egypt; g Department of Chemistry, College of Science, Qassim University 51452 Buraidah Saudi Arabia; h Department of Physics, College of Science, Qassim University Buraydah 51452 Saudi Arabia; i Department of Physics, Emerson University Multan 60000 Pakistan arslan.ashfaq@eum.edu.pk

## Abstract

This study investigates a tin-based perovskite solar cell (PSC) incorporating an inorganic hole transport layer, examined through simulations with the SCAPS simulator. The chosen CuZnSn(Se_1−*x*_S_*x*_) compound emerges as a promising candidate for the hole transport layer, allowing for a tunable band gap *via* adjustments to the S/(S + Se) ratio. The band gap varies from 0.95 eV for Cu_2_ZnSnSe_4_ to 1.5 eV for Cu_2_ZnSnS_4_, achieved through strategic valence band offset engineering at the MAPbI_3_/CuZnSn(Se_1−*x*_S_*x*_) interface. However, achieving an optimal Valence Band Offset (VBO) at MASnI_3_/CuZnSn(Se_1−*x*_S_*x*_) remains challenging yet crucial for realizing high-performance Perovskite Solar Cells. The device efficiency is systematically optimized by manipulating the S content, resulting in a noteworthy Power Conversion Efficiency of 18.29%. Furthermore, it is uncovered that a carefully selected VBO (0.22 eV) is achieved with the CZTSe_0.4_S_0.6_ hole transport layer, contributing significantly to the improved performance of the PSC. These findings underscore the importance of precise engineering in achieving optimal device properties for advanced solar energy conversion applications.

## Abstract 2

Tin-based perovskite solar cells with a CuZnSn(Se_1−*x*_S_*x*_) HTL are optimized *via* SCAPS simulation. Tunable VBO enables 18.29% efficiency, with CZTSe_0.4_S_0.6_ showing ideal band alignment for enhanced photovoltaic performance.

## Graphical abstract

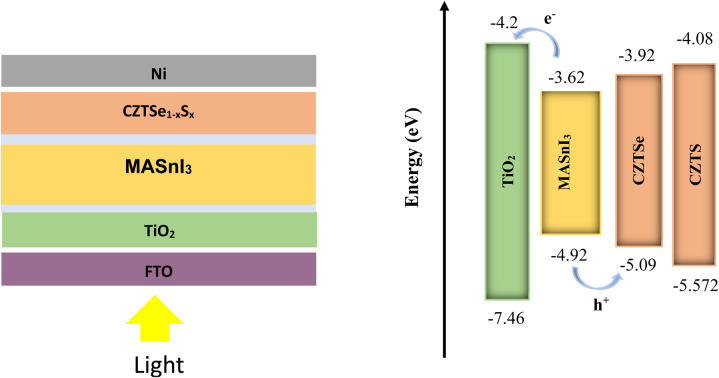

## 1 Introduction

In recent years, there has been substantial progress in advancing perovskite solar cells (PSCs), a trend that began with the initial report in 2009.^1^ A lot of work has gone into producing solar cells with greater efficiency, with perovskite materials emerging as one of the most promising alternatives for future developments. The power conversion efficiency (PCE) of hybrid halide perovskite has increased dramatically, from 3.8% to an astounding 25.2%, which justifies the material's increased attention on a global scale.^2,3^ Metal halide perovskites, or PSCs, are represented by the formula ABX_3_, where A stands for non-bonding monovalent cations, such as methylammonium (MA)^+^, formamidinium (FA)^+^, and cesium (Cs)^+^; B represents octahedral divalent ions, usually Pb^2+^; and X is a monoanionic ion, usually from the halide group (Cl^−^, Br^−^, I^−^, or a mixture).

Compared to more known technologies such as CdTe, CIGS, and silicon solar cells, this photovoltaic (PV) breakthrough has been achieved rather quickly. Characteristics such as an appropriate band gap, high absorption coefficient, ambipolar charge-carrier transport, small exciton binding energy, long diffusion length, defects tolerance, low fabrication cost, and low effective carrier masses are responsible for PSCs success.^4,5^

Despite the remarkable accomplishments of PSCs, challenges remain for their broader adoption. Lead (Pb), a toxic element, risks human health and the natural environment.^6^ Additionally, MAPbI_3_ experiences reduced performance due to poor stability when exposed to moisture and sunlight.^7^ To address these issues, various nontoxic metals such as copper, bismuth, germanium, antimony, and tin have been explored as substitutes for Pb in PSCs.^8,9^ Sn has been identified as the most promising substitution for improving PSC performance.

MASnI_3_, with its narrow band gap of 1.3 eV compared to MAPbI_3_, exhibits a similar valency. The slight reduction in the radius of Sn^2+^ (1.35 Å) compared to Pb^2+^ (1.49 Å) allows for the replacement of Pb^2+^ with Sn^2+^ while maintaining the perovskite structure.^10^ The Goldschmidt tolerance factor and octahedral factor play a critical role in assessing the structural stability and feasibility of forming perovskite phases, thereby serving as essential criteria for the rational design and selection of high-performance perovskite materials in solar cell applications.^11^ Sn^2+^ substitution adheres to coordination, causes minimal lattice constant perturbation, and maintains ionic size and charge balance.^12,13^ Tin-based, environmentally friendly perovskite materials hold the potential to enhance device performance and stability. However, tin-based PSCs face challenges, including sensitivity to oxygen leading to rapid oxidation of Sn^2+^ into Sn^4+^, as well as the self-doping effect and rapid crystallization rate during solution preparation, resulting in current losses and reduced power conversion efficiency (PCE).^14,15^

Numerous techniques have emerged to enhance the performance of MASnI_3_-based devices. For instance, adopting a n–i–p device configuration with mesoscopic TiO_2_ and Spiro-OMeTAD as the hole transport material has achieved a PCE of 6.4%.^16^ In 2019, MASnI_3_ achieved a PCE of 7.19% through a normal device by employing cation exchange approaches.^17^ The proper selection of electron transport materials (ETM) and hole transport materials (HTM) is crucial for improving the efficiency, reproducibility, and stability of solar cell. However, the use of organic materials such as Spiro-OMeTAD, PATT, and PEDOT:PSS poses challenges due to potential degradation and high costs, hindering the commercialization of perovskites.^18^

Inorganic copper-based HTLs, including CuSbS_2_ and CuSCN, have been investigated for their suitable band gap and long-term stability.^19^ However, the performance of devices using these materials falls short compared to their organic counterparts. To further enhance the efficiency of PSCs with in-organic HTLs, it is crucial to identify other materials with appropriate energy band positions, inherent properties, excellent chemical stability, and high conductivity.^20^

Copper-based materials possess chemical stability, high conductivity, and hole transport mobility, making them suitable candidates as HTLs in PSCs. Cu_2_ZnSnS_4_ is an environmentally friendly, nontoxic, and abundant material with a high absorption coefficient of 10^5^ cm^−1^, an heightened energy band gap of 1.5 eV, and excellent stability, making it viable for use in low price devices. While the maximum achieved PCE of CZTS is 9.6%, the alloying of Cu_2_ZnSn(Se_1−*x*_S_*x*_)_4_ compound has demonstrated superior performance, reaching 12.6% efficiency with a tunable band gap.^21,22^ Notably, CZTSe_1−*x*_S_*x*_ has not only been explored as a potential light absorber in thin-film solar cells but has also shown promise as an HTL in PSCs, achieving an efficiency of 22.77%.^23^ CZTSe_1−*x*_S_*x*_ can be synthesized using a nanoparticle ink method, eliminating the need for high-temperature sulfuration and enabling its use at lower temperatures. A study in 2016 investigated the band level alignment and valence band position influence of CZTS and CZTSe nanoparticles ink as HTL on the performance of PSCs.^24^

This manuscript introduces the novel concept of utilizing CZTSe_1−*x*_S_*x*_ as an HTL in MASnI_3_ PSCs through Solar Cell Capacitance Simulator (SCAPS), marking the first time this device modeling approach has been considered. The traditional structure of PSCs is examined and discussed, focusing on the valence band offset between the perovskite light absorber and CZTSe_1−*x*_S_*x*_, as well as the band gap of the HTL controlled through composition engineering of the CZTSe_1−*x*_S_*x*_ compound. The effects of these factors on the *IV* characteristics and efficiency parameters are thoroughly investigated, leading to an optimized power conversion efficiency achieved with a specific S composition. At CZTSe_0.4_S_0.6_ composition, a notable efficiency of 17.29% is attained. Further optimization of carrier concentration, defect density, and diffusion length of tin-based perovskite in CZTSe_0.4_S_0.6_ results in an enhanced PCE of 17.34%.

## 2 Device configuration

**Fig. 1:**
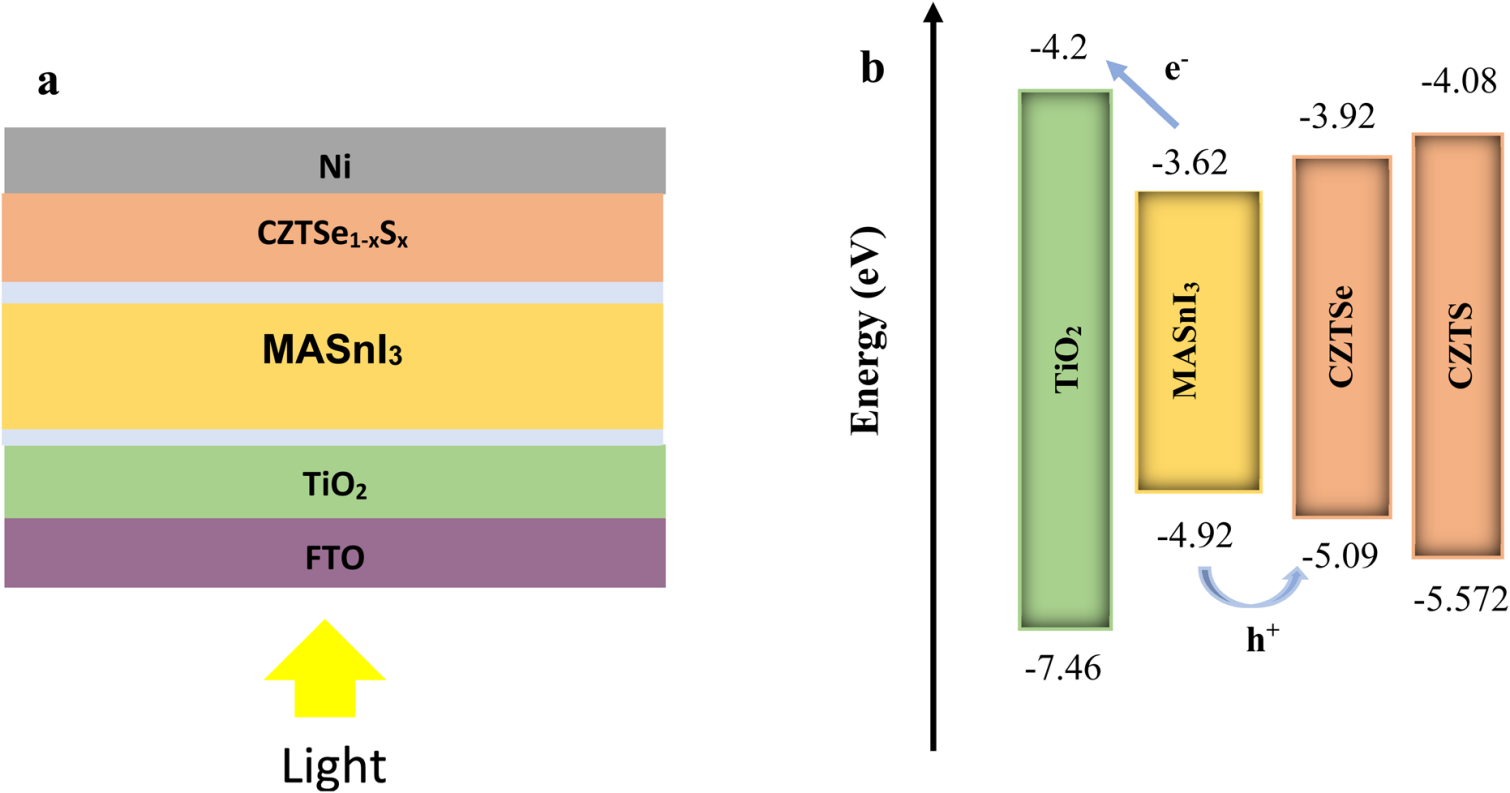
(a) Architecture of modelling device (b) energy level alignment.

The schematic representation of the simulated structure and the energy level alignment of the device structure are illustrated in Fig. 1. The cell configuration comprises a SLG substrate/FTO (used as TCO)/TiO_2_ (serving as ETL)/MASnI_3_ as the absorber/CZTSe_1−*x*_S_*x*_ (employed as HTL)/Ni (functioning as the back contact), as depicted in Fig. 1(a). The energy levels and electron affinities of the main layers are presented in Fig. 1(b).

The simulation of the device is conducted using SCAPS 3.310, a software developed by the Department of Electronics and Information Systems (ELIS) at the University of Gent.^25^ The simulation is performed under 1 Sun illumination with an incident power density of 1 kW m^−2^ at a temperature of 300 K. SCAPS utilizes well-established equations from literature,^26^ including the continuity equation, Poisson's equation, and electron/hole transport equations, to model various recombination mechanisms for solar cell simulation.

Table 1 presents fundamental parameters obtained from experimental and published data for the HTL, ETL, and FTO, as documented in sources. To represent the optical behavior of each layer accurately, the absorption coefficients for all active layers were incorporated. The absorption coefficient of MASnI_3_ was modeled using the Tauc relation with a pre-factor *A*_α_ = 10^5^, while the absorption profiles of TiO_2_ and CZT(Se_1−*x*_S_*x*_) were extracted from reported experimental literature.^27,30,31^ The effective density states for valence and conduction bands is established at 1.8 × 10^19^ and 2.2 × 10^18^ cm^−3^, respectively, with the exception of the ETL.^32^ The thermal velocities of holes and electrons are set at 1 × 10^7^ cm s^−1^. For accurate representation, MASnI_3_ is assigned an electron carrier density of 2.0 × 10^16^ cm^−3^.

**Table 1:** The initial input parameters of the simulated device^27–29^

Parameters	FTO	TiO_2_	MASnI_3_	Spiro-OMeTAD	CZTSe	CZTS
*ε*_r_	9	10	8.2	3.0	10	10
*χ* (eV)	4	4	4.17	2.45	4.35	4.5
*E*_g_ (eV)	3.5	3.2	1.3	3.0	0.95	1.5
*N*_c_ (cm^−3^)	2.2 × 10^18^	2 × 10^17^	1.0 × 10^18^	2.2 × 10^18^	2.2 × 10^18^	2.2 × 10^18^
*N*_v_ (cm^−3^)	1.8 × 10^19^	6 × 10^17^	1.0 × 10^18^	1.8 × 10^19^	1.8 × 10^19^	1.8 × 10^19^
*μ*_n_ (cm^−2^ V^−1^ s^−1^)	20	100	1.6	2 × 10^−4^	60	100
*μ*_p_ (cm^−2^ V^−1^ s^−1^)	10	25	1.6	2 × 10^−4^	20	25
*N*_a_ (cm^−3^)	0	0	0	2 × 10^18^	5 × 10^16^	5 × 10^16^
*N*_d_ (cm^−3^)	2 × 10^19^	2 × 10^19^	2 × 10^16^	10	10	10
*v*_e_ (cm s^−1^)	1 × 10^7^	1 × 10^7^	1 × 10^7^	1 × 10^7^	1 × 10^7^	1 × 10^7^
*v*_h_ (cm s^−1^)	1 × 10^7^	1 × 10^7^	1 × 10^7^	1 × 10^7^	1 × 10^7^	1 × 10^7^
*d* (nm)	50	50	450	200	200	200
*N*_t_ (cm^−3^)	1 × 10^15^	1 × 10^14^	2.5 × 10^13^	1 × 10^14^	1 × 10^14^	1 × 10^14^

To account for interface recombination, two Interface Defect Layers (IDLs) are introduced between TiO_2_/perovskite and MASnI_3_/HTL. These IDLs share identical parameters with MASnI_3_ for consistency.

To achieve a carrier lifetime of 1 ns in the absorber layer, a defect density of 2.5 × 10^13^ cm^−3^ is presumed, aligning with the theoretical range of 1 ns to 4 ns. Table 2 compiles information on interface defects and absorber defects. Front optical filtration employs Transmission Solar Glass, and the back contact metal work function is set to 5.3 eV.^33^

**Table 2:** The input parameters of defect in MASnI_3_ and interface of layers

Parameters	MASnI_3_	TiO_2_/IDL	IDL2/HTL
Defect type	Neutral	Neutral	Neutral
Capture cross section for electrons/holes (/cm^2^)	2 × 10^−14^	1 × 10^−18^	1 × 10^−18^
	2 × 10^−14^	1 × 10^−17^	1 × 10^−19^
Energetic distribution	Gaussian	Single	Single
Energy level w.r.t *E*_v_ (above *E*_v_, eV)	0.65	0.07	0.32
Characteristics energy/eV	0.1	—	—
Total density/cm^−3^	Variable	1 × 10^9^	1 × 10^9^

The band gap (*E*_g_) and electron affinity (*χ*) of the CZTSe_1−*x*_S_*x*_ HTL are adjusted according to the sulfur content (*x*). Previous studies indicated that the *E*_g_ values for CZTS (*x* = 1) and CZTSe (*x* = 0) were 1.5 and 0.95 eV, respectively. The determination of *E*_g_ and *χ* for CZTSe_1−*x*_S_*x*_ at various sulfur concentrations (*x*) is accomplished using equations from the literature.^28^

## 3 Results and discussion

**Fig. 2:**
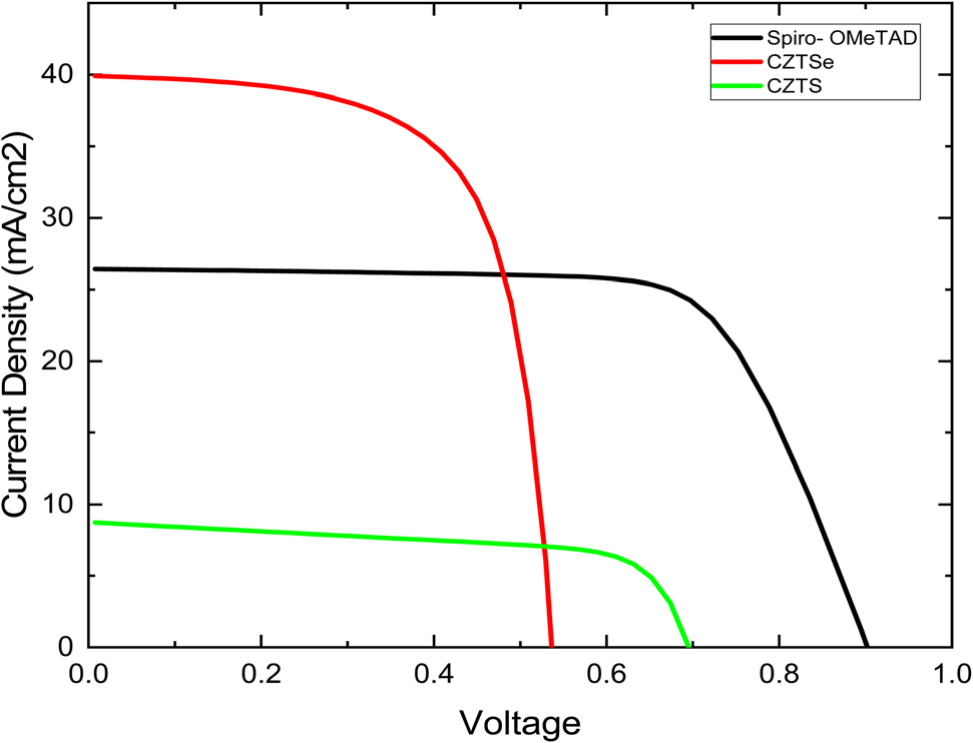
The numerical simulated *J*–*V* properties of different HTL.

The simulation primarily focuses on the tin-based perovskite structure with Spiro-OMeTAD as the Hole Transport Layer (HTL), aiming to compare CZTSe and CZTS, respectively. Fig. 2 illustrates the numerical simulated *J*–*V* curve of the reference solar cell. The reference cell, utilizing Spiro-OMeTAD, exhibits a Power Conversion Efficiency of 23.36%, *V*_oc_ of 0.93 V, *J*_sc_ of 31.60 mA cm^−2^, and a FF of 79.99%. These values strongly correlate with reported experimental and theoretical data for high-efficiency PSCs with performance exceeding 20%.^34,35^

In addition to the reference cell, the study explores CZTSe and CZTS as HTLs, keeping all other parameters constant except for those related to the HTL. The simulated *J*–*V* properties with CZTSe HTL show a *V*_oc_ of 0.52 V, *J*_sc_ of 39.88 mA cm^−2^, FF of 66.28%, and an efficiency of 13.95%. Notably, all efficiency parameters in this simulation are lower than those with Spiro-OMeTAD HTL in tin-based perovskite solar cells, yet these results align with previously reported experimental outcomes. The simulations are conducted with a thickness of 200 nm and a total defect density of 5 × 10^16^ cm^−3^ for both CZTSe and CZTS.

Our simulation reveals that CZTSe, as an HTL, demonstrates a power conversion efficiency comparable to Spiro-OMeTAD in tin-based PSCs. This similarity is attributed to the approximately aligned band structures of MASnI_3_/Spiro-OMeTAD and MASnI_3_/CZTSe interfaces.

**Fig. 3:**
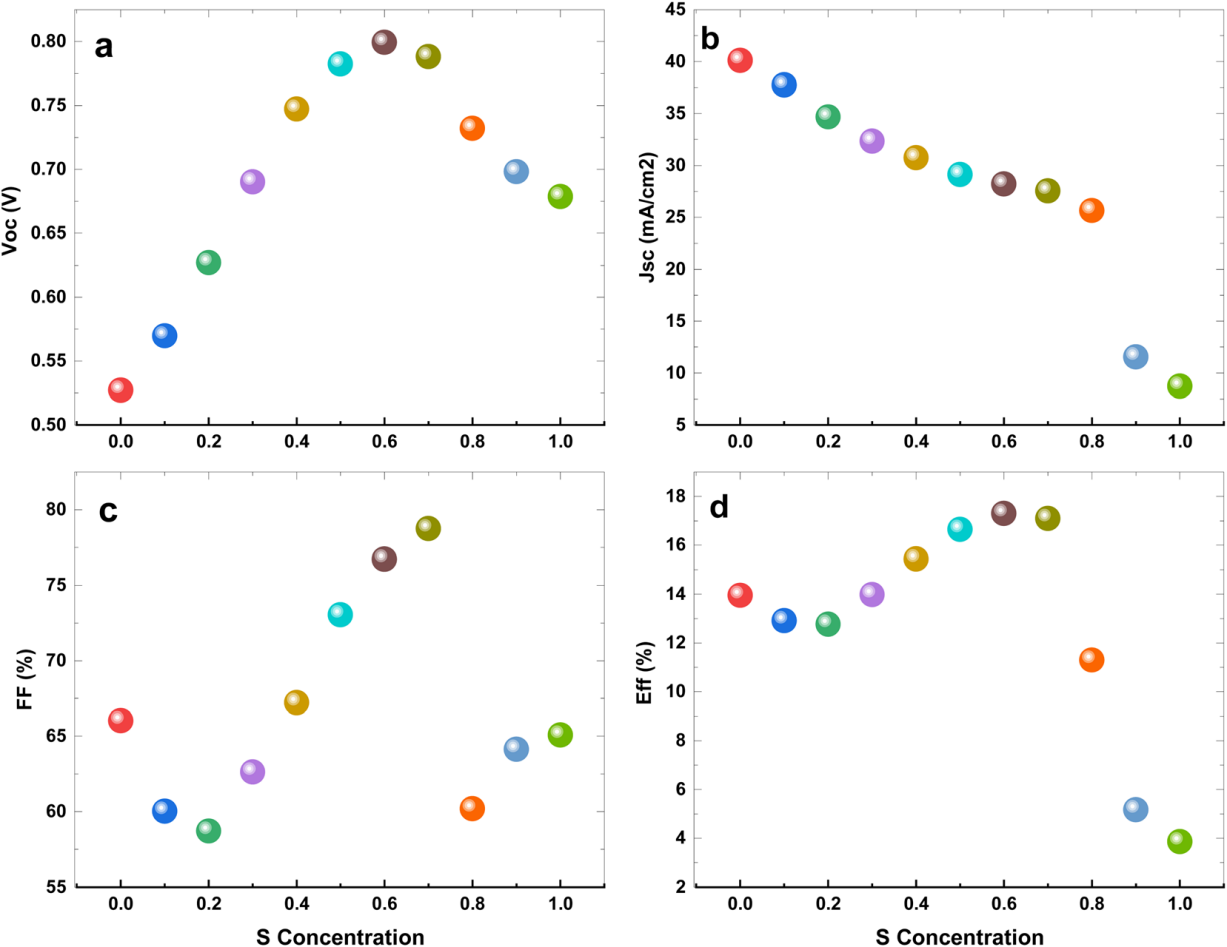
The variation in the performance of the IV parameters with the different S concentration in CZTSe_1−*x*_S_*x*_ as HTL in structure.

The CZTSe alloys possess the capability of a tunable energy band gap. Therefore, employing a configuration engineering method allows for the investigation of the CZTSe_1−*x*_S_*x*_ compound and the optimization of band alignment at interfaces, consequently influencing PCE in tin-based devices. The changes in performance of the IV parameters with different sulfur concentrations in CZTSe_1−*x*_S_*x*_ as the Hole Transport Layer (HTL) is depicted in Fig. 3.

Tin-based PSCs with CZTSe as the HTL exhibit relatively lower performance IV parameters. With the addition of a small fraction of S to CZTSe, *J*_sc_ experiences a slight decrease, followed by a more substantial decrease. As the S concentration increases up to approximately 0.6, *V*_oc_, *J*_sc_, and *η* show an increase, while FF initially decreases before starting to increase again, as shown in Fig. 3(c). Beyond *x* = 0.7, a significant decrease in all parameters is observed. Therefore, optimized performance is observed at *x* = 0.6.

At the adjusted of the sulfur content, the tin-based PSCs demonstrate a *V*_oc_ of 0.79 V, *J*_sc_ of 28.20 mA cm^−2^, FF of 76.71%, and PCE of 17.29%. This efficiency is in correspondence with Spiro-OMeTAD-based lead-free PSCs.

**Fig. 4:**
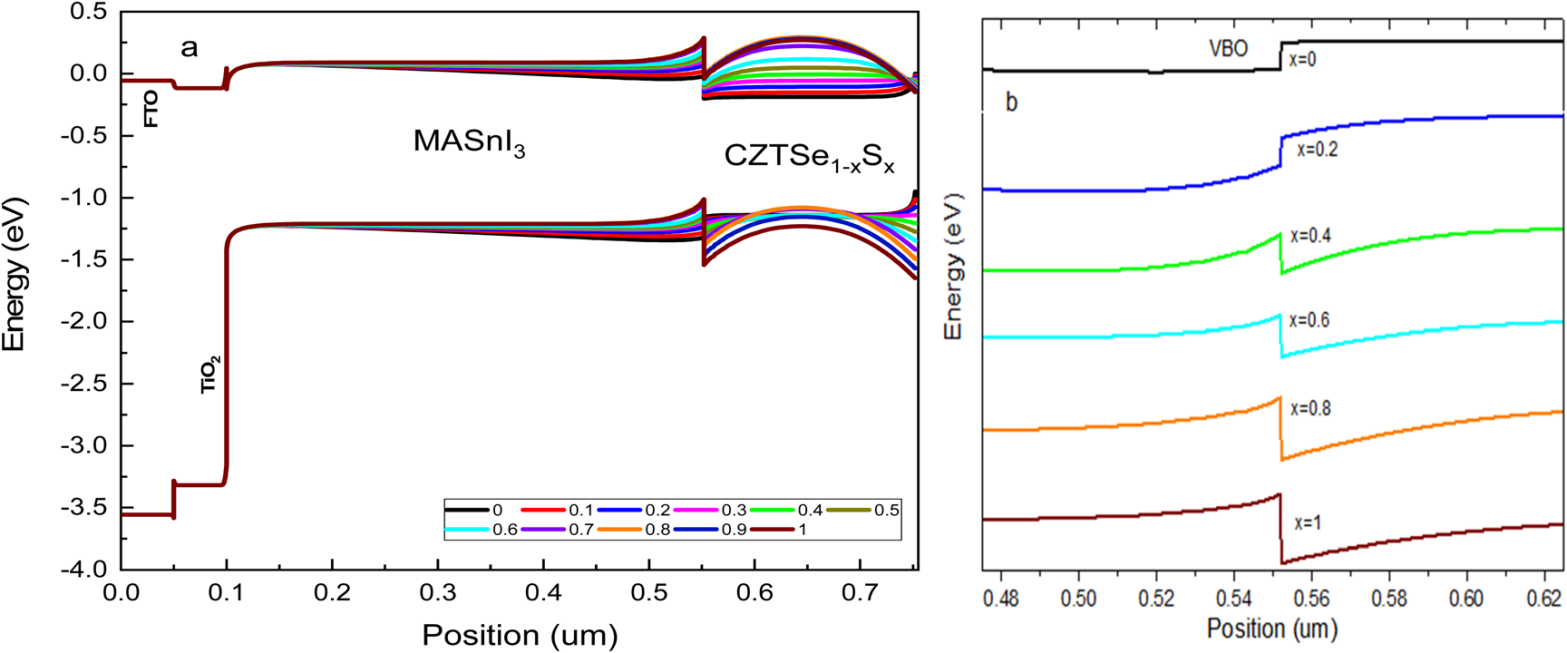
(a) Energy band diagram of tin based PSCs with CZTSe_1−*x*_S_*x*_ HTL (b) MASnI_3_/HTL valence band offset.

The S/(S + Se) ratio serves to control the band gap (*E*_g_) and electron affinity (*χ*) of the CZTSe_1−*x*_S_*x*_ Hole Transport Layer (HTL). Through composition engineering, the Conduction Band Offset (CBO) and Valence Band Offset (VBO) at the interface of MASnI_3_/CZTSe_1−*x*_S_*x*_ can be practically manipulated. The band diagram of MASnI_3_ with CZTSe_1−*x*_S_*x*_ HTL is presented in Fig. 4(a). To facilitate the hole charge transportation from the tin-based absorber layer to the HTL, the valence energy band level of the HTL is usually slightly lower than the conduction band level of MASnI_3_, leading to a reduction in the Built-in Potential (*V*_bi_). It is related that a small negative VBO can result in a low *V*_oc_. CZTSe shows a −0.17 eV VBO and the lowest *V*_bi_ (−0.35 V), which corresponds to the smallest *V*_oc_ in Fig. 3(a).

**Fig. 5:**
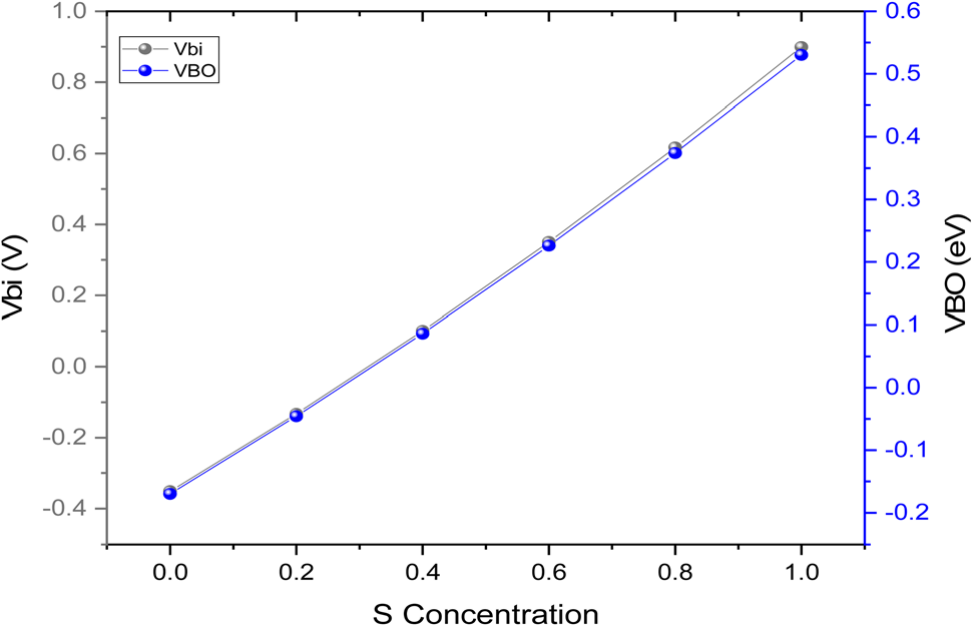
The change of VBO and *V*_bi_ with different S content in CZTSe_1−*x*_S_*x*_ HTL.

The VBO of CZTSe_1−*x*_S_*x*_ consistently shifts to deeper energy levels, resulting in values of VBO and *V*_bi_ transitioning from negative to positive with the addition of sulfur concentration in CZTSe, as indicated in Fig. 4b and 5. A positive (+ive) VBO is most favorable for reducing charge carrier recombination.^36^ The complete valence energy band alignment at the MASnI_3_/CZTSe_1−*x*_S_*x*_ interface is illustrated in Fig. 4(a and b) with a distinctly vertical transfer. The fluctuation in the valence energy band position of CZTSe_1−*x*_S_*x*_ alters the *V*_bi_ in the absorber layer.

The *V*_bi_ is defined as 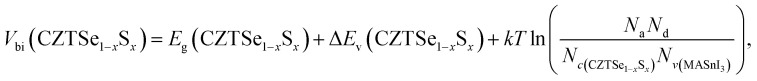 and the VBO = *χ*(CZTSe_1−*x*_S_*x*_) + *E*_g_(CZTSe_1−*x*_S_*x*_) − *χ*(MASnI_3_) − *E*_g_(MASnI_3_). The values of VBO and *V*_bi_ are attained and depicted in Fig. 5. CZTS shows the maximum VBO (0.53 eV) and *V*_bi_ (0.89 V) as the VBO becomes deeper with increasing sulfur content, leading to an increase in *V*_bi_. In our study, tin-based PSCs with CZTSe_0.6_S_0.4_ or CZTSe_0.4_S_0.6_ HTLs show a +ive VBO of 0.08 eV and 0.22 eV, respectively. From Fig. 5, it can be inferred that tin-based PSC with CZTSe_0.4_S_0.6_ HTL demonstrates sufficient *V*_bi_, which significantly participate to the larger *V*_oc_ examined in Fig. 3(a).

**Fig. 6:**
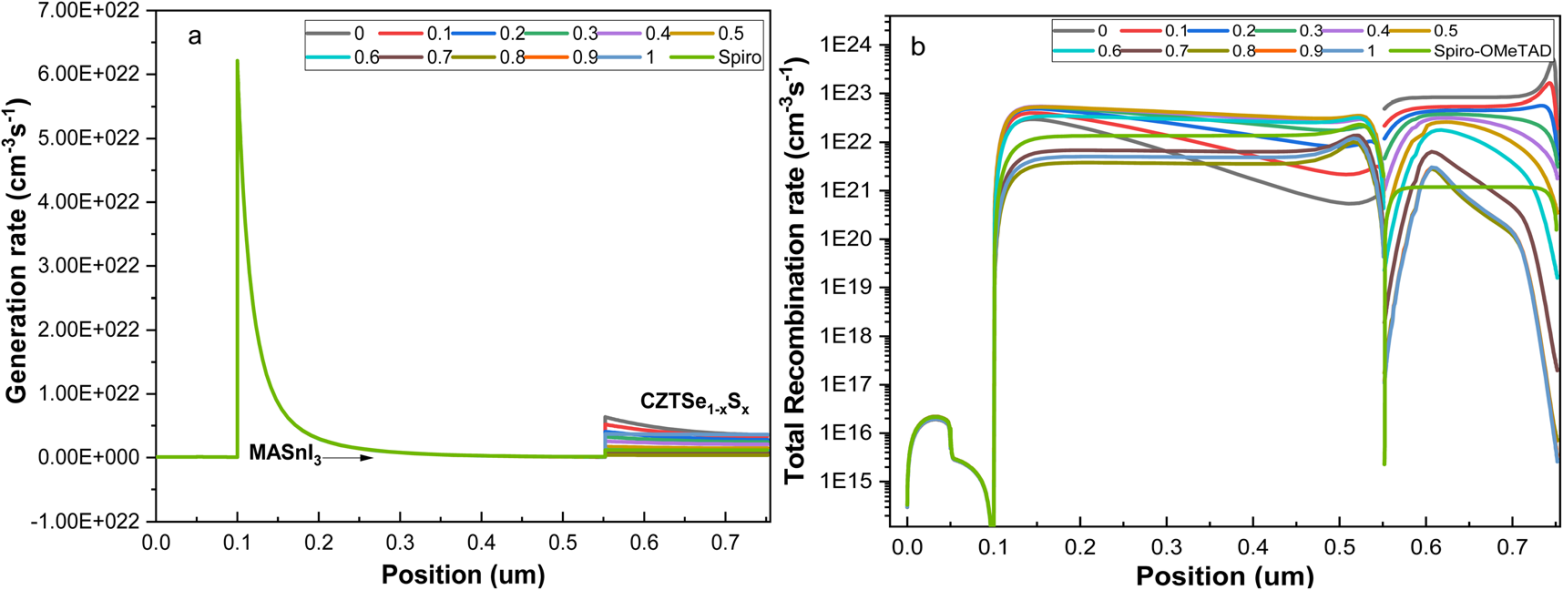
The recombination rate and charge carrier generation of the PSCs with CZTSe_1−*x*_S_*x*_ HTL.

The overall rate of generation and recombination in tin-based PSCs with CZTSe_1−*x*_S_*x*_ is depicted in Fig. 6. With a thickness of 450 nm for MASnI_3_, the majority of incident light is absorbed, allowing only a small portion to be transmitted, given its large range of the absorption co-efficient (10^4^–10^5^ cm^−3^). As seen in Fig. 6(a), carrier generation primarily appears in the MASnI_3_ layer. The suitable band gap of CZTSe_1−*x*_S_*x*_ in the range of 0.95–1.5 eV impacts the generation rate and recombination rate of the charge carriers for the transmitted light.

When CZTSe_1−*x*_S_*x*_ is employed as the Hole Transport Layer (HTL), maximum charge carrier generation was examined in CZTSe. The charge carrier generation rate decreases as the sulfur content increases, attributed to the widening band gap. The interface of MASnI_3_/CZTSe_1−*x*_S_*x*_, a potential barrier hinders the motion of electrons from the HTL to MASnI_3_ due to the Conduction Band Offset (CBO). Simultaneously, the *V*_bi_ in the perovskite layer propels photon-generated majority charge carriers toward the CZTSe_1−*x*_S_*x*_, facilitating easy recombination with already available minority carriers in the CZTSe_1−*x*_S_*x*_.^37^

Hence, the total recombination rate in CZTSe_1−*x*_S_*x*_ is noted to be less than Spiro-OMeTAD. For certain concentrations, the recombination rate is particularly small due to the reduced accessibility of the recombined electrons, driven by the high sulfur concentration and an appropriate Valence Band Offset (VBO). Among the CZTSe_1−*x*_S_*x*_ compositions, CZTSe_0.4_S _0.6_ exhibits a slightly lower total recombination rate.

**Fig. 7:**
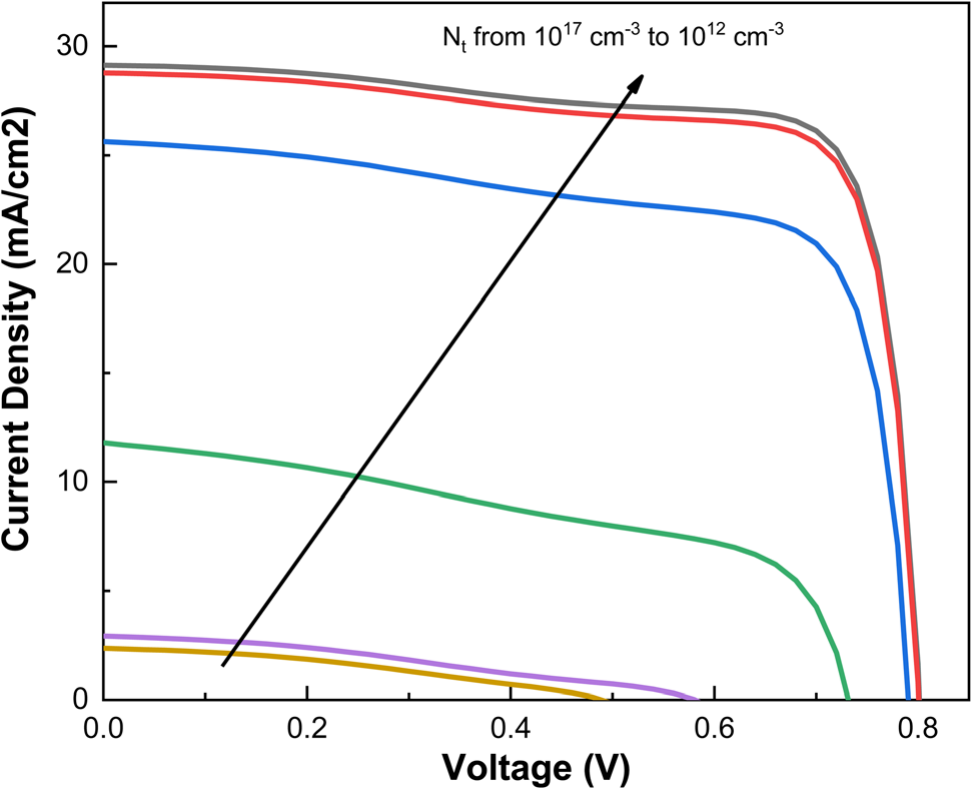
Variation in *I*–*V* curves with increasing the values of *N*_t_ in CZTSe_0.4_S_0.6_.

The defect density (*N*_t_) of MASnI_3_ is adjusted to 2.5 × 10^13^ cm^−3^, aligning with the carrier diffusion length of 0.9 μm based on earlier numerical simulated investigations of prime structure. To investigate the impact of *N*_t_ further, we varied *N*_t_ from 10^12^–10^17^ cm^−3^ and illustrated the change of *I*–*V* curves with *N*_t_ in Fig. 7. The cell demonstrates a substantial performance improvement with a decrease in *N*_t_ in tin-based perovskite, consistent with simulations of lead-based perovskite.^38^ A lower *N*_t_ results in better photovoltaic performance, achieving *V*_oc_ of 0.80 V, *J*_sc_ of 29.13 mA cm^−2^, FF of 78.31%, and PCE of 18.29%.

**Fig. 8:**
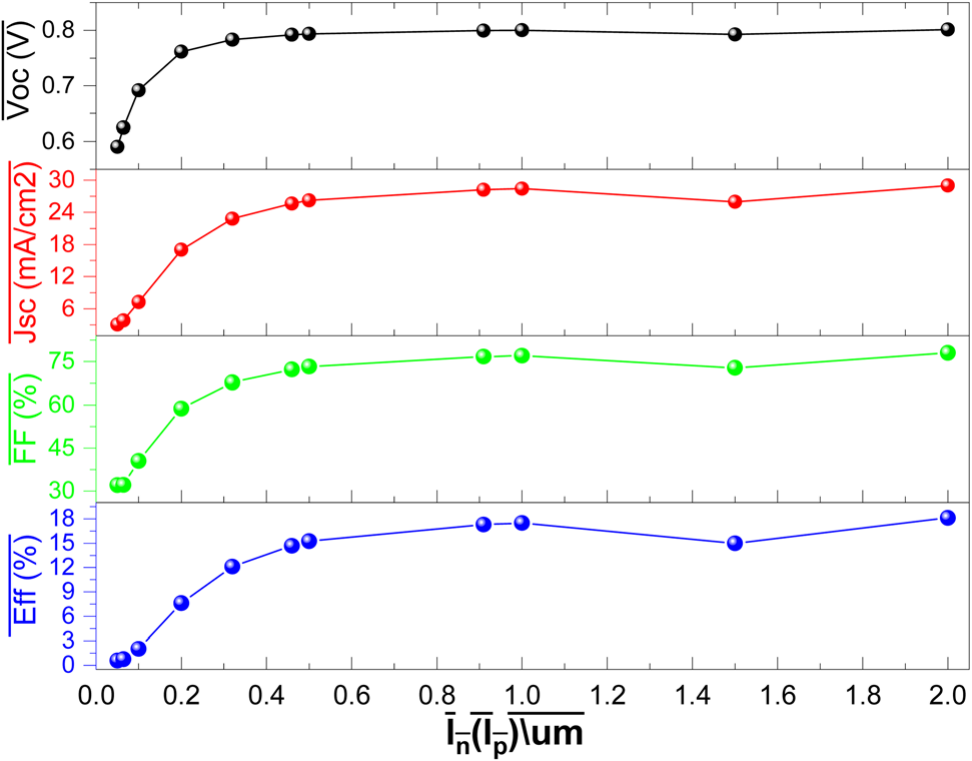
Variation in device efficiency, with rising diffusion length in MASnI_3_/CZTSe_0.4_S_0.6_.

Experimental literature indicates that Sn-based PSCs exhibits favorable charge-transport characteristics. To delve deeper into the effect of *N*_t_ on the device, we examine the impact of *N*_t_ on the charge carrier diffusion length (*L*) in Fig. 8, based on 
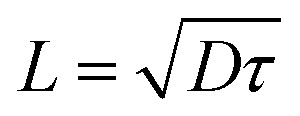
 and 
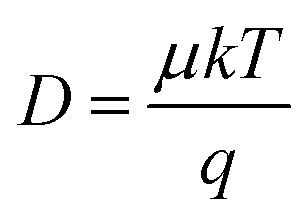
, where μ represents carrier mobility and is used to determine the diffusion length of carriers. Lower *N*_t_ values correspond to longer diffusion lengths (*L*), which contributes to the enhancement of cell performance.

Considering the influence of *N*_t_ and *l*_n_(*l*_p_), the devices performance parameters are optimum when *N*_t_ is as low as 2.491 × 10^13^ cm^−3^ (resulting in a *l*_n_(*l*_p_) of 0.9 μm), and the absorber layer thickness is 450 nm. This substantial performance improvement is attributed to the increased *l*_n_(*l*_p_) associated with the reduction in *N*_t_. Table 3 provides a comparative summary of MASnI_3_-based perovskite solar cells reported in the literature along with the current simulated device. In this study, a PCE of 18.29% was achieved for the MASnI_3_/CuZnSn(Se_1−*x*_S_*x*_) heterostructure, demonstrating the role of optimized absorber thickness, defect passivation, and favorable band alignment through interface engineering. Notably, an experimental absorption model was employed in our simulation, resulting in more realistic and experimentally relevant efficiency values. In contrast, many reported studies rely on idealized absorption assumptions, often overestimating performance. Although some literature devices show higher PCEs (*e.g.*, 25.05% with CuI HTL, 24.28% using BaTiO_3_/CuO interfaces), they typically incorporate more complex or less stable components. Our proposed structure offers a cost-effective, stable, and environmentally friendly architecture, providing a promising foundation for experimental fabrication and further optimization of high-efficiency, lead-free perovskite solar cells.

**Table 3:** Comparative performance of MASnI_3_-based perovskite solar cells reported in the literature and the material and interface engineering

Absorber material	Device structure	PCE (%)	References
MASnI_3_	MASnI_3_/CuZnSn(Se_1−*x*_S_*x*_)	18.29	This work
MASnI_3_	PCBM/MASnI_3_/CuI	25.05	39
MASnI_3_	MASnI_3_/CuSCN	20.17	40
MASnI_3_	p-P3HT/p-MASnI_3_	22.46	41
MASnI_3_	BaTiO_3_/MASnI_3_/CuO	24.28	42
MASnI_3_	BaTiO_3_/MASnI_3_/MASnBr_3_	24.09	42

## 4 Conclusion

This study explores a Sn-based PSCs with an inorganic HTL, utilizing the SCAPS simulator. The compound Cu_2_ZnSn(Se_1−*x*_S_*x*_)_4_ emerges as a promising candidate for the HTL in tin-based PSCs, offering a tunable band gap through changing the S/(S + Se) ratio. This range spans from 0.95 eV for Cu_2_ZnSnSe_4_ to 1.5 eV for Cu_2_ZnSnS_4_, achieved through appropriate VBO engineering at the MAPbI_3_/CuZnSn(Se_1−*x*_S_*x*_) interface. Achieving a proper VBO at MASnI_3_/CuZnSn(Se_1−*x*_S_*x*_) is challenging but crucial for obtaining high-performance PSCs. Optimization of solar cell performance is carried out by adjusting the S concentration, resulting in PCE of 18.29%. Additionally, it is revealed that an appropriate VBO (0.22 eV) is achieved with the CZTSe_0.4_S_0.6_ hole transport layer, contributing to enhanced PSC performance.

## Data availability

Data available on suitable request.

## Conflicts of interest

There are no conflicts to declare.
